# Mortality and detailed characteristics of pre-ICU qSOFA-negative patients with suspected sepsis: an observational study

**DOI:** 10.1186/s13613-018-0389-3

**Published:** 2018-04-03

**Authors:** Izumi Nakayama, Junichi Izawa, Hideyuki Mouri, Tetsuhisa Kitamura, Junji Shiotsuka

**Affiliations:** 1 Intensive Care Unit, Department of Internal Medicine, Okinawa Chubu Hospital, 281 Miyazato, Uruma, Okinawa 904-2293 Japan; 2 Intensive Care Unit, Department of Anesthesiology, The Jikei University School of Medicine, 3-19-18 Nishi-Shinbashi, Minato-ku, Tokyo, 105-8471 Japan; 3 The Center for Critical Care Nephrology, Clinical Research, Investigation, and Systems Modeling of Acute Illness Center, Department of Critical Care Medicine, University of Pittsburgh School of Medicine, Pittsburgh, PA 15213 USA; 4 Department of Anesthesiology and Critical Care, Jichi Medical University, Saitama Medical Center, 1-847 Amanuma, Oomiya-ku, Saitama, Saitama 330-8503 Japan; 5 Division of Environmental Medicine and Population Sciences, Department of Social and Environmental Medicine, Graduate School of Medicine, Osaka University, 1-1 Yamada-oka, Suita, Osaka 565-0871 Japan

**Keywords:** Intensive care unit, Critical care, Bacteremia, Sepsis, quick Sequential Organ Failure Assessment (qSOFA) score, Infection, Mortality

## Abstract

**Background:**

Recent studies have suggested that quick Sequential Organ Failure Assessment (qSOFA) scores have limited utility in early prognostication in high-mortality populations. The purpose of this study was to investigate the association between pre-ICU qSOFA scores and in-hospital mortality among patients admitted to the ICU with suspected sepsis. This study also aimed to describe detailed clinical characteristics of qSOFA-negative (< 2) patients.

**Methods:**

This single center, observational study, conducted in a Japanese tertiary care teaching hospital between May 2012 and June 2016, enrolled all consecutive adult patients admitted to the ICU with suspected sepsis. We assessed pre-ICU qSOFA scores with the most abnormal vital signs during the 24-h period before ICU admission. The primary outcome was in-hospital mortality censored at 90 days. We analyzed the association between pre-ICU qSOFA scores and in-hospital mortality.

**Results:**

Among 185 ICU patients with suspected sepsis, 14.1% (26/185) of patients remained qSOFA-negative at the time of ICU admission and 29.2% (54/185) of patients died while in hospital. In-hospital mortality was similar between the groups (qSOFA-positive [≥ 2]: 30.2% [48/159] vs qSOFA-negative: 23.1% [6/26], *p* = 0.642). The Cox proportional hazard regression model revealed that being qSOFA-positive was not significantly associated with in-hospital mortality (adjusted hazard ratio 1.35, 95% confidence interval 0.56–3.22, *p* = 0.506). Bloodstream infection, immunosuppression, and hematologic malignancy were observed more frequently in qSOFA-negative patients.

**Conclusions:**

Among ICU patients with suspected sepsis, we could not find a strong association between pre-ICU qSOFA scores and in-hospital mortality. Our study suggested high mortality and bacterial diversity in pre-ICU qSOFA-negative patients.

**Electronic supplementary material:**

The online version of this article (10.1186/s13613-018-0389-3) contains supplementary material, which is available to authorized users.

## Background

Early identification and interventions have been shown to improve sepsis outcomes [1, 2]. Recently, the quick Sequential Organ Failure Assessment (qSOFA) score was developed to promptly identify infected patients at risk of mortality. The original study showed that qSOFA-positive (≥ 2) patients had a 3- to 14-fold increase in in-hospital mortality compared to qSOFA-negative (< 2) patients [3]. With its simple and repeatedly measurable property, qSOFA has had a promising role in providing a more effective triage for infected patients [4].

However, recent studies have suggested that qSOFA has limited utility in early prognostication in high-mortality populations. One study showed that almost one-half of patients with infection remained qSOFA-negative even at the time of ICU admission [5]. In studies enrolling patients admitted to the ICU, the mortality of qSOFA-negative patients was greater than 10% [5–9]. Thus, the usefulness of qSOFA scores in high-risk populations has remained controversial.

We hypothesized that, for patients with suspected sepsis requiring ICU admission, the prognostic impact of qSOFA-positive was small. The purpose of this study was to investigate the association between pre-ICU qSOFA scores, assessed during the 24-h period before ICU admission, and in-hospital mortality among patients admitted to the ICU with suspected sepsis. Furthermore, we described detailed clinical characteristics of qSOFA-negative patients including clinical diagnosis, primary sites of infection, causative organisms, and comorbidities. Given this description, we aimed to disclose features of patients whose risk of mortality was difficult to estimate using qSOFA.

## Methods

### Study design, setting, and patients

This was an observational study conducted at the Okinawa Chubu Hospital, a tertiary care teaching hospital with 550 hospital beds and 14 ICU beds in Japan, between May 2012 and June 2016. The hospital institutional review board approved the study protocol (H28-14). Because of the retrospective approach of this study and de-identification of personal data, the board waived the need for informed consent.

We examined data of all adult (≥ 18 years) patients who were admitted to the ICU between May 2012 and June 2016. We identified consecutive patients with suspected sepsis through the following inclusion criteria: the documentation of the reason for ICU admission as ‘bacteremia,’ ‘sepsis,’ ‘severe sepsis,’ or ‘septic shock’ in the ICU register. Each documentation was based on the clinical judgment as having a severe infection requiring ICU admission. Two attending physicians reviewed the patient data and agreed on the clinical suspicion of infection. We excluded patients with cardiac arrest prior to ICU admission because we did not expect an additional predictive value of qSOFA in these patients.

### Data collection

Data for analyses including age, sex, chronic health conditions, location prior to ICU admission, vital signs and qSOFA scores before ICU admission, the presence of rigor (‘shaking chills’), primary site of infection, type of organisms, length of ICU stay, the prevalence of bacteremia and in-hospital mortality were collected from patient records. According to a previous report from our institution [10], we routinely classified the qualitative degree of rigor (‘chills’) as follows: ‘mild chills,’ feeling cold with the need for an outer jacket; ‘moderate chills,’ feeling very cold with the need for a thick blanket; and ‘shaking chills,’ a profound chill with generalized involuntary bodily shaking, even under a thick blanket. Physicians were instructed to record the degree of chills when they suspected bacteremia in daily practice. We described the primary site of infection as bloodstream, respiratory, gastrointestinal, neurological, genitourinary, or musculoskeletal infection based on the clinical context. Bloodstream infection was defined as blood culture-positive infection including infective endocarditis, bacteremia from an unknown origin and catheter-related bacteremia. The primary infection site showed the following organism types, namely gram-negative bacterial infection, gram-positive bacterial infection, polymicrobial infection or fungal infection. Illness severity was assessed using the Acute Physiology and Chronic Health Evaluation (APACHE) II [11] and the Sequential Organ Failure Assessment (SOFA) scores [12] with the most abnormal measurements recorded during the first 24-h period after ICU admission (Additional file 1: Fig. S1). We used the worst SOFA scores and defined sepsis as a SOFA score of ≥ 2 according to the Sepsis-3 definition [4].

### Measurement of the main exposure factors (Additional file 1: Fig. S1)

The qSOFA score had three criteria, assigning one point for alteration in mental status (Glasgow Coma Scale < 15), systolic blood pressure ≤ 100 mm, Hg or respiratory rate ≥ 22/min [4]. We evaluated pre-ICU qSOFA scores with the most abnormal vital signs at the time of clinical deterioration during the 24-h period before ICU admission. We set this time window to evaluate the performance value of pre-ICU admission qSOFA scores in prognosticating high-risk patients before ICU transfer. We also aimed to avoid the effect of therapeutic interventions during the ICU stay on qSOFA scores. According to a previous study, we defined qSOFA-positive or qSOFA-negative as a qSOFA score of ≥ 2 or < 2, respectively [3]. We also evaluated pre-ICU systemic inflammatory response syndrome (SIRS) with the most abnormal measurements during the 24-h period before ICU admission. SIRS-positive was defined as two or more of the following: temperature > 38 or < 36 °C, heart rate > 90 beats/min, respiratory rate > 20 breaths/min, or arterial carbon dioxide pressure < 32 mm Hg, white blood cell count > 12,000/μL or < 4000/μL [13]. In addition, we evaluated qSOFA scores and SIRS at the exact moment of ICU arrival using the first measurements of vital signs just after ICU admission.

### Outcome measures

The primary outcome measure was in-hospital mortality, which was defined as any cause of death censored at 90 days after ICU admission. Other outcomes included the length of ICU stay, ICU stay ≥ 3 days, bacteremia and in-hospital mortality censored at 28 days after ICU admission. We defined bacteremia as 2 sets of blood culture with the same microorganism or 1 set of blood culture with bacteria, except for possible contaminated resources involving Coagulase-negative *Staphylococci*, *Corynebacterium* species, *Propionibacterium* species, *Bacillus* species, *Aerococcus* species, and *Micrococcus* species [14, 15].

### Statistical analysis

Continuous data are presented as medians with interquartile range (IQR) and compared using the Mann–Whitney *U* test. Categorical data are presented as proportions and compared using a *Chi*-*squared* test or Fisher’s exact test when appropriate. We used Kaplan–Meier plots to describe the survival between qSOFA-positive and qSOFA-negative patients and compared the survival curves with the log-rank test. As the primary analysis, the Cox proportional hazard regression model was used to assess the association between being qSOFA-positive before ICU admission and in-hospital mortality censored at 90 days after ICU admission. The hazard ratio (HR) and 95% confidence interval (CI) were calculated. The following variables were incorporated into the primary multivariable models: age, the presence of rigor (‘shaking chills’), prior location to the ICU, and chronic health condition with immunosuppression. In the Kaplan–Meier description and the Cox regression analysis, if survival hospital discharge occurs within 90 days after ICU admission, we dealt with it as censoring. We also estimated the performance of pre-ICU qSOFA, pre-ICU SIRS, qSOFA at ICU arrival, and SIRS at ICU arrival in predicting sepsis by the Sepsis-3 and in-hospital mortality censored at 90 days. The crude risk ratios (RRs) with 95% CI and area under receiver operating characteristics (AUROC) were calculated. We used qSOFA scores and SIRS scores as continuous variables for the calculation of AUROCs. All statistical analyses were performed using R (The R Foundation for Statistical Computing, ver.3.2.4) and EZR (Saitama Medical Center, Jichi Medical University, ver.1.32), which is a graphical user interface for R [16]. All tests were two-tailed; *p* values of less than 0.05 were regarded as statistically significant.

## Results

**Fig. 1:**
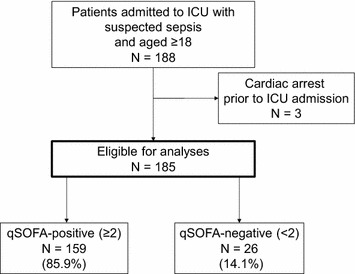
Flow of patients admitted to the ICU with suspected sepsis from 2012 to 2016. *ICU* denotes intensive care unit, *qSOFA* quick Sequential Organ Failure Assessment

The patient flow diagram is presented in Fig. 1. We extracted 188 patients who were admitted to the ICU with suspected sepsis. After excluding 3 patients with prior cardiac arrest, we enrolled 185 patients for our analyses. At least 2 sets of blood cultures were obtained from all participants. The median age was 67 (IQR 57–79), and 61.1% (113/185) of patients were from the emergency room (ER) and 33.5% (62/185) of patients had at least one chronic health condition. Among 185 patients, 85.9% (159/185) were qSOFA-positive and 89.7% (166/185) were SIRS-positive before ICU admission. The median APACHE II and SOFA scores were 21 (IQR 17–28) and 9 (IQR 5–11), respectively. In total, 91.9% (170/185) of patients fulfilled the Sepsis-3 definition, 53.0% (98/185) had positive blood culture, and 29.2% (54/185) died in hospital within 90 days after ICU admission.

Patient demographics, characteristics at the presentation of infection, and characteristics after ICU admission are presented in Table 1. While qSOFA-positive patients were presented with more deranged vital signs, qSOFA-negative patients were more frequently from the ward (qSOFA-positive: 35.8% [57/159] vs qSOFA-negative: 57.7% [15/26], *p* = 0.050) and more frequently had shaking chills (qSOFA-positive: 27.7% [44/159] vs qSOFA-negative: 53.8% [14/26], *p* = 0.011). Hematologic malignancy (qSOFA-positive: 5.7% [9/159] vs qSOFA-negative: 15.4% [4/26], *p* = 0.090) and immunosuppression (qSOFA-positive: 5.7% [9/159] vs qSOFA-negative: 15.4% [4/26], *p* = 0.090) were also observed more frequently in qSOFA-negative patients. The degree of organ dysfunction was similar between the groups in relation to respiration, coagulation, and liver and renal components of the SOFA scores.

**Table 1:** Characteristics before and after ICU admission in patients with suspected sepsis

	qSOFA-positive (≥ 2)	qSOFA-negative (< 2)	*p* value
	(*N* = 159)	(*N* = 26)	
*Demographics*			
Age (median [IQR])	67 [57–79]	68 [63–75]	0.791
Male (%)	95 (59.7%)	16 (61.5%)	1.000
Chronic health condition (%)			
Metastatic cancer	7 (4.4%)	1 (3.8%)	1.000
Chronic dialysis	22 (13.8%)	4 (15.4%)	0.767
Hepatic failure	15 (9.4%)	2 (7.7%)	1.000
Chronic respiratory failure	4 (2.5%)	1 (3.8%)	0.535
Hematologic malignancy	9 (5.7%)	4 (15.4%)	0.090
Immunosuppression	9 (5.7%)	4 (15.4%)	0.090
*Characteristics at the presentation of infection*			
Location prior to ICU admission (%)			0.050
Emergency room	102 (64.2%)	11 (42.3%)	
General ward	57 (35.8%)	15 (57.7%)	
Vital signs before ICU admission (median [IQR])			
Systolic blood pressure, mm Hg	82 [70–96]	105 [79–120]	0.004
Respiratory rate,/min	28 [24–30]	20 [20–24]	< 0.001
Glasgow coma scale	13 [9–15]	15 [15–15]	< 0.001
Heart rate,/min	116 [101–132]	110 [88–119]	0.047
Body temperature, Celsius	38.2 [37.2–39.1]	38.2 [37.1–39.2]	0.997
Shaking chills (%)	44 (27.7%)	14 (53.8%)	0.011
*Characteristics after ICU admission*			
APACHE II (median [IQR])	22 [17–29]	20 [15–24]	0.092
SOFA score (median [IQR])	9 [6–12]	6 [3–8]	0.001
SOFA respiration	2 [1–3]	1 [0–3]	0.112
SOFA coagulation	1 [0–2]	1 [0–2]	0.256
SOFA liver	0 [0–1]	0 [0–0]	0.296
SOFA central nervous system	1 [0–3]	0 [0–0]	< 0.001
SOFA renal	1 [0–3]	1 [0–2]	0.887
SOFA cardiovascular	3 [1–4]	1 [0–3]	< 0.001
Lactate, mmol/L (median [IQR])	2.5 [1.5–5.3]	1.5 [0.9–3.5]	0.075

qSOFA scores were assessed with the most abnormal vital signs during the 24-h period before the ICU admission. SOFA and APACHE II scores were calculated with the most abnormal measurements taken during the first 24-h period after the ICU admission

*APACHE II* Acute Physiology and Chronic Health Evaluation II, *ICU* intensive care unit, *IQR* interquartile range, *qSOFA* quick Sequential Organ Failure Assessment, *SOFA* Sequential Organ Failure Assessment

**Fig. 2:**
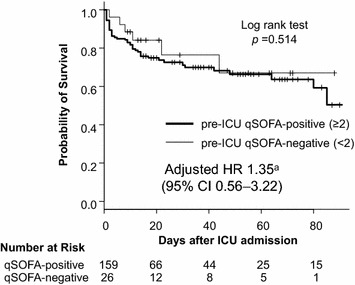
Kaplan–Meier curves of in-hospital mortality censored at 90 days stratified as pre-ICU qSOFA-positive or qSOFA-negative. ^a^Adjusted for age, the presence of rigor (‘shaking chills’), prior location to the ICU and chronic health condition with immunosuppression. *CI* confidence interval, *HR* hazard ratio, *ICU* intensive care unit, *qSOFA* quick Sequential Organ Failure Assessment. The vertical tick marks on the curves denote censoring due to survival discharge

The outcomes of qSOFA-negative patients were similar to those of qSOFA-positive patients (Fig. 2 and Table 2). The primary outcome, in-hospital mortality censored at 90 days after ICU admission, was not significantly different between the groups (qSOFA-positive: 30.2% [48/159] vs qSOFA-negative: 23.1% [6/26], *p* = 0.642). The other outcomes, ICU length of stay (qSOFA-positive: 3 [2–6] vs qSOFA-negative: 3 [2–5], *p* = 0.787), bacteremia (qSOFA-positive: 53.5% [85/159] vs qSOFA-negative: 50.0% [13/26], *p* = 0.833), and 28-day mortality (qSOFA-positive: 25.2% [40/159] vs qSOFA-negative: 19.2% [5/26], *p* = 0.627) were also similar between the groups. The Kaplan–Meier plots of survival showed no significant difference between the groups (*p* = 0.514). The Cox proportional hazard regression model revealed that pre-ICU qSOFA-positive was not significantly associated with in-hospital mortality (adjusted HR 1.35, 95% CI 0.56–3.22, *p* = 0.506).

**Table 2:** ICU stay and in-hospital mortality in ICU patients with suspected sepsis

	qSOFA-positive (≥ 2)	qSOFA-negative (< 2)	*p* value
	(*N* = 159)	(*N* = 26)	
ICU length of stay (median [IQR])	3 [2–6]	3 [2–5]	0.787
ICU stay ≥ 3 days (%)	94 (59.1%)	15 (57.7%)	1.000
Bacteremia (%)	85 (53.5%)	13 (50.0%)	0.833
In-hospital mortality (%)			
28-day mortality	40 (25.2%)	5 (19.2%)	0.627
90-day mortality	48 (30.2%)	6 (23.1%)	0.642

qSOFA scores were assessed with the most abnormal vital signs taken during the 24-h period before the ICU admission

In-hospital mortality was defined as any cause of death censored at 28 days or at 90 days after the ICU admission

*ICU* intensive care unit, *IQR* interquartile range, *qSOFA* quick Sequential Organ Failure Assessment

Detailed microbiological results are presented in Table 3. Among primary sites of infection, bloodstream infection was more frequent in qSOFA-negative patients (qSOFA-positive: 18.2% [29/159] vs qSOFA-negative: 30.8% [8/26], *p* = 0.094). Among identified organisms, *Staphylococcus aureus* infection was more frequent in qSOFA-negative patients (qSOFA-positive: 8.8% [14/159] vs qSOFA-negative: 15.4% [4/26], *p* = 0.290). Of 26 qSOFA-negative patients, the most common site of infection was bloodstream infection (30.8% [8/26]), followed by genitourinary infection (23.1% [6/26]). Among qSOFA-negative patients who died in the hospital, all the patients had at least one chronic health condition.

**Table 3:** Microbiological results in ICU patients with suspected sepsis

	qSOFA-positive (≥ 2)	qSOFA-negative (< 2)	*p* value
	(*N* = 159)	(*N* = 26)	
Primary site of infection (%)			
Bloodstream	29 (18.2%)	8 (30.8%)	0.094
Respiratory	31 (19.5%)	3 (11.5%)	0.573
Gastrointestinal	25 (15.7%)	4 (15.4%)	0.770
Neurological	2 (1.3%)	0 (0.0%)	NA
Genitourinary	39 (24.5%)	6 (23.1%)	1.000
Musculoskeletal	19 (11.9%)	1 (3.8%)	0.476
Other	14 (8.8%)	4 (15.4%)	0.290
Type of organisms (%)			
Gram-negative bacterial infection	66 (41.5%)	11 (42.3%)	
*Escherichia coli*	30 (18.9%)	5 (19.2%)	1.000
*Klebsiella pneumoniae*	12 (7.5%)	1 (3.8%)	0.697
*Pseudomonas aeruginosa*	7 (4.4%)	0 (0.0%)	NA
Gram-positive bacterial infection	32 (20.1%)	6 (23.1%)	
*Staphylococcus aureus*	14 (8.8%)	4 (15.4%)	0.290
*Streptococcus pneumoniae*	9 (5.7%)	0 (0.0%)	NA
*Streptococcus* species	4 (2.5%)	1 (3.8%)	0.535
Polymicrobial infection	20 (12.6%)	4 (15.4%)	0.752
Fungal infection	4 (2.5%)	0 (0.0%)	NA
Not specified	37 (23.3%)	5 (19.2%)	0.803

qSOFA scores were assessed with the most abnormal vital signs taken during the 24-h period before the ICU admission

Bloodstream infection was defined as blood culture-positive infection including infective endocarditis, bacteremia from an unknown origin and catheter-related bacteremia

*ICU* intensive care unit, *NA* not applicable, *qSOFA* quick Sequential Organ Failure Assessment

The performance of qSOFA and SIRS in predicting sepsis and mortality is shown in Table 4. The association between pre-ICU qSOFA or pre-ICU SIRS and in-hospital mortality censored at 90 days was not significant (qSOFA crude RR 1.38, 95% CI 0.62–2.74, AUROC 0.511; SIRS crude RR 0.92, 95% CI 0.45–1.85, AUROC 0.521). On the other hand, qSOFA at ICU arrival was significantly associated with in-hospital mortality censored at 90 days (crude RR 1.78, 95% CI 1.09–2.89, AUROC 0.586).

**Table 4:** Performance of qSOFA and SIRS in predicting sepsis and mortality

	Sepsis by Sepsis-3 definition			In-hospital mortality		
	*n*/*N* (%)	Crude risk ratio (95% CI)	AUROC	n/*N* (%)	Crude risk ratio (95% CI)	AUROC
Pre-ICU qSOFA			0.711			0.511
qSOFA-positive (≥ 2)	149/159 (93.7%)	1.16 (0.96–1.41)		48/159 (30.2%)	1.38 (0.62–2.74)	
qSOFA-negative (< 2)	21/26 (80.8%)	1.00 (ref)		6/26 (23.1%)	1.00 (ref)	
Pre-ICU SIRS			0.710			0.521
SIRS-positive (≥ 2)	155/166 (93.4%)	1.18 (0.93–1.50)		48/166 (28.9%)	0.92 (0.45–1.85)	
SIRS-negative (< 2)	15/19 (78.9%)	1.00 (ref)		6/19 (31.6%)	1.00 (ref)	
qSOFA at ICU arrival			0.624			0.586
qSOFA-positive (≥ 2)	92/98 (93.9%)	1.05 (0.96–1.14)		36/98 (36.7%)	1.78 (1.09–2.89)	
qSOFA-negative (< 2)	78/87 (89.7%)	1.00 (ref)		18/87 (20.7%)	1.00 (ref)	
SIRS at ICU arrival			0.709			0.541
SIRS-positive (≥ 2)	133/139 (95.7%)	1.19 (1.03–1.38)		41/139 (29.5%)	1.04 (0.62–1.77)	
SIRS-negative (< 2)	37/46 (80.4%)	1.00 (ref)		13/46 (28.3%)	1.00 (ref)	

Pre-ICU qSOFA and SIRS scores were assessed with the most abnormal vital signs taken during the 24-h period before the ICU admission. qSOFA and SIRS scores at ICU arrival were assessed with the first measurements just after ICU admission

Sepsis was defined according to the Sepsis-3 definition. In-hospital mortality was defined as any cause of death censored at 90 days after the ICU admission

*AUROC* area under receiver operating characteristics, *CI* confidence interval, *ICU* intensive care unit, *qSOFA* quick Sequential Organ Failure Assessment, *SIRS* systemic inflammatory response syndrome

## Discussion

Our study suggested that the prognostic impact of pre-ICU qSOFA, assessed during the 24-h period before ICU admission, was small among patients with suspected sepsis (HR 1.35, 95% CI 0.56–3.22). In this study, comprised of high-mortality (29.2%) patients, 14% (26/185) of patients remained qSOFA-negative even at the time of ICU admission. Moreover, the difference in in-hospital mortality was small (qSOFA-positive: 30.2% vs qSOFA-negative: 23.1%, *p* = 0.642). Importantly, our study suggested that the risk of mortality in patients with bloodstream infection, immunosuppression or hematologic malignancy would be difficult to estimate using qSOFA scores. The results of our study may provide important implications for clinicians in early prognostication of patients with suspected sepsis and for developers of sepsis screening systems.

Among patients with suspected infection outside the ICU, qSOFA scores had greater prognostic accuracy than SIRS [3]. Since 1992, SIRS has gained widespread acceptance as the clinical definition of sepsis [13]. However, the specificity of SIRS ≥ 2 was too low and 70–90% of ICU patients, including non-infected patients, attained SIRS ≥ 2 during their ICU stay [17]. Along with the development of a new definition for sepsis, the qSOFA score has been generated to guide bedside clinicians in identifying infected patients at risk of in-hospital mortality or longer ICU stay [3]. The original study showed that qSOFA-positive patients had a 3- to 14-fold increase in in-hospital mortality compared to qSOFA-negative patients when qSOFA scores were assessed during the 72-h period around the onset of infection. Further external validation studies have shown that qSOFA scores had greater prognostic accuracy than SIRS among patients presenting to the ER [18–20].

However, recent studies have suggested that qSOFA has limited utility in early prognostication in high-mortality populations. In a retrospective analysis of a large adult ICU patient database, qSOFA assessed during the first 24-h following ICU admission had little additional predictive value for mortality over SIRS [6]. In recent studies consisting of patients admitted to the ICU or patients in the ward, in-hospital mortality of qSOFA-negative patients was higher (13.6–17.4%) compared to mortality in studies consisting of ER patients [5–9]. Therefore, qSOFA was assumed to have limited performance value in prognosticating high-risk patients. Importantly, qSOFA scores assessed after ICU admission were likely to have been affected with therapeutic interventions such as vasopressors and sedative agents [3, 6]. Therefore, pre-ICU qSOFA scores assessed before ICU admission have been evaluated [21].

We focused on patients with suspected sepsis requiring ICU admission and evaluated pre-ICU qSOFA scores assessed during the 24-h period before ICU admission. Our results raise a question as to why the association between pre-ICU qSOFA-positive and mortality was weaker (HR 1.35, 95% CI 0.56–3.22) than that observed in previous studies [3, 18]. To address this question, it is to be noted, first, that our patients were judged as having a severe infection by treating physicians before enrollment. Physicians detected signs of severe infection based not only on vital sign abnormalities such as qSOFA components but also on clinical diagnosis, primary sites of infection, presumed causative organisms and patient comorbidities [22, 23]. Also, some experts have questioned the sensitivity of qSOFA because qSOFA would remain negative until life-threatening organ dysfunction has developed [24]. A previous study showed that qSOFA remained negative even at the time of ICU transfer in one-half of infected patients [5]. In our study, the association between qSOFA and mortality became significant only after ICU admission (Pre-ICU qSOFA: crude RR 1.38, 95% CI 0.62–2.74; qSOFA at ICU arrival: crude RR 1.78, 95% CI 1.09–2.89). Physicians might have detected the risk of further clinical deterioration before qSOFA was determined as positive. As a result, the association between pre-ICU qSOFA and mortality would have attenuated. Our results suggested that qSOFA had little additional predictive value for mortality over clinical judgment (Fig. 2, Table 4). Second, we presented 90-day mortality instead of 28-day mortality. Recent studies have shown that patients with sepsis had increasing mortality beyond the standard 28-day mortality and that the use of long-term outcomes had been postulated to infer the full impact of sepsis [25]. Our study represented long-term outcomes of infected patients requiring ICU admission.

In addition to investigating the association between qSOFA-positive and mortality, we described detailed characteristics in qSOFA-negative patients to disclose features of patients whose risk of mortality was difficult to estimate using qSOFA scores (Table 1, 3). The characteristics, which were more frequently found in qSOFA-negative patients, were hematologic malignancy, immunosuppression, bloodstream infection, and *Staphylococcus aureus* infection. Among 8 bloodstream infections in qSOFA-negative patients, 37.5% (3/8) had chronic dialysis, and 25% (2/8) had hematologic malignancy. We think that the history of comorbidities alerted physicians of further deterioration and prompted physicians to consider ICU transfer before qSOFA scores turned positive. A variety of infections were presented with qSOFA-negative patients in our study. Indeed, we often experienced infective endocarditis, catheter-related bacteremia, pyelonephritis, and bacterial pneumonia in qSOFA-negative patients. Of note, all the patients who died in the qSOFA-negative group had at least one chronic health condition. In these patients, primary sites of infection and comorbidities would be additional useful information for early prognostication.

Our study had several limitations. First, our study was conducted in a single center with a small number of patients. As a result, only 54 in-hospital deaths were observed and the CI for our primary analyses was wide (HR 1.35, 95% CI 0.56–3.22). It is possible that we failed to find an association between pre-ICU qSOFA and in-hospital mortality due to the small sample size. Because no study focused on pre-ICU qSOFA at the time we planned the study, it was difficult to estimate a priori sample size. Second, because we did not observe all the infected patients presented to the ER or the ward, it is possible that we did not accurately estimate the association between qSOFA and mortality in patients not requiring ICU admission. The generalizability of the result of this study might have been attenuated. Currently, however, only a few studies have focused on pre-ICU qSOFA scores and on qSOFA-negative, infected ICU patients [21]. The results of our study provide an important basis for further prospective studies investigating the role of qSOFA in triage decisions for ICU admission. Third, we did not use uniform criteria for ICU admission. The threshold of ICU transfer in each patient largely depends on physicians and hospital-beds availability. Nevertheless, the median APACHE II scores (21, IQR 17–28), SOFA scores (9, IQR 5–11), and mortality (29.2%) of our patients were higher than those of related studies [3, 6, 18] or than in recent multicenter studies enrolling patients with early septic shock (mortality 18%) [26]. Thus, our results reflected the performance value of qSOFA in high-risk populations. Last, due to the retrospective nature of our study, the frequency of qSOFA variable measurements was not standardized. The pre-ICU qSOFA scores in our study were based on the worst vital signs that were obtainable during the 24-h period before ICU admission. There were no missing data regarding qSOFA scores.

## Conclusion

In this observational study, among patients admitted to the ICU with suspected sepsis, we could not find a strong association between pre-ICU qSOFA scores and in-hospital mortality. We described high mortality and bacterial diversity in pre-ICU qSOFA-negative patients. Besides qSOFA scores, primary sites of infection and comorbidities may provide additional useful information for early prognostication in high-risk populations.

## Additional file

**Additional file 1: Fig. S1.** Pre-ICU qSOFA and SIRS were evaluated with the most abnormal measurements during the 24-hour period before ICU admission. qSOFA and SIRS at ICU arrival were evaluated with the first measurements recorded just after ICU admission. SOFA and sepsis by the Sepsis-3 definition were evaluated with the most abnormal measurements during the first 24-hour period after ICU admission. ICU denotes intensive care unit; qSOFA, quick Sequential Organ Failure Assessment; SIRS, systemic inflammatory response syndrome; SOFA, Sequential Organ Failure Assessment.

## Abbreviations

APACHE II: Acute Physiology and Chronic Health Evaluation II
AUROC: area under receiver operating characteristic
CI: confidence interval
ER: emergency room
HR: hazard ratio
ICU: intensive care unit
IQR: interquartile range
qSOFA: quick Sequential Organ Failure Assessment
RR: risk ratio
SIRS: systemic inflammatory response syndrome
SOFA: Sequential Organ Failure Assessment
