# Supplementary figures and images for: Mortality and detailed characteristics of pre-ICU qSOFA-negative patients with suspected sepsis: an observational study
Source: Ann Intensive Care. 2018 Apr 3;8:44. doi: 10.1186/s13613-018-0389-3 (PMC5882475; doi:10.1186/s13613-018-0389-3)

## Slide 1
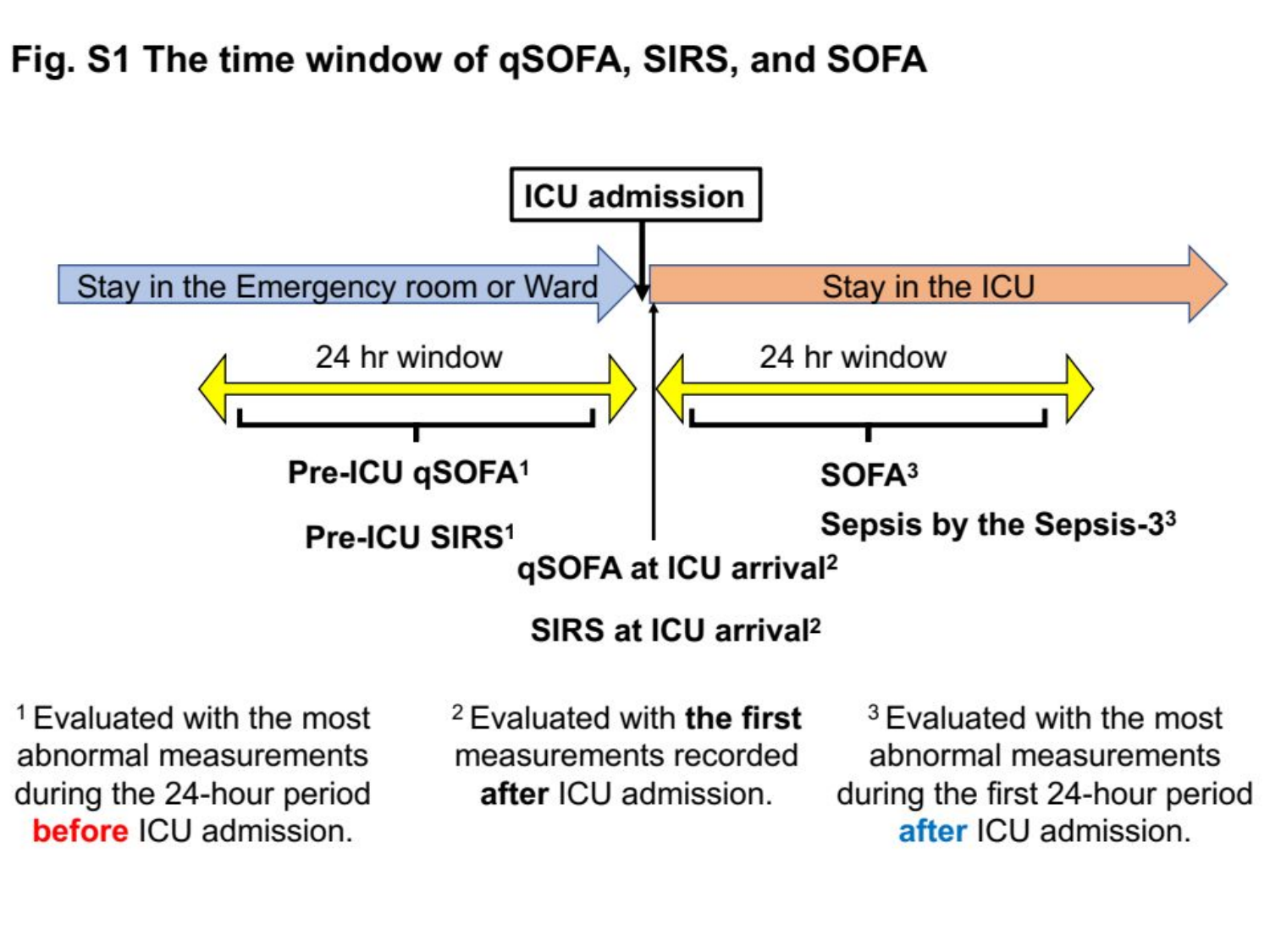

#

Supplement: Supplementary file 1 — Additional file 1: Fig. S1. Pre-ICU qSOFA and SIRS were evaluated with the most abnormal measurements during the 24-hour period before ICU admission. qSOFA and SIRS at ICU arrival were evaluated with the first measurements recorded just after ICU admission. SOFA and sepsis by the Sepsis-3 definition were evaluated with the most abnormal measurements during the first 24-hour period after ICU admission. ICU denotes intensive care unit; qSOFA, quick Sequential Organ Failure Assessment; SIRS, systemic inflammatory response syndrome; SOFA, Sequential Organ Failure Assessment. [file 13613_2018_389_MOESM1_ESM.pptx]
